# Supplementary figures and images for: Mechanical force regulates the inhibitory function of PD-1
Source: EMBO Rep. 2026 Feb 25;27(7):1789–812. doi: 10.1038/s44319-026-00715-6 (PMC13076993; doi:10.1038/s44319-026-00715-6)

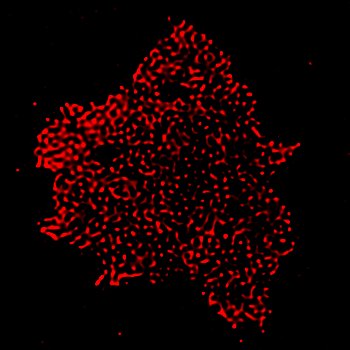

Supplement: Supplementary file 6 — Source data Fig. 4 [file 44319_2026_715_MOESM6_ESM.zip › Figure 4/Figure 4F/hPDl1-WT-cropped-mcherry.jpg]

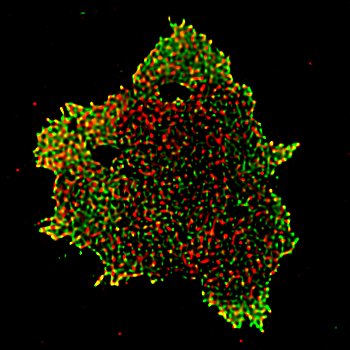

Supplement: Supplementary file 6 — Source data Fig. 4 [file 44319_2026_715_MOESM6_ESM.zip › Figure 4/Figure 4F/hPDl1-WT-cropped-merge.jpg]

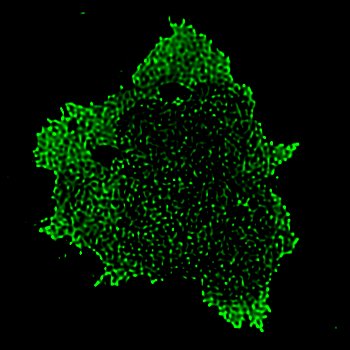

Supplement: Supplementary file 6 — Source data Fig. 4 [file 44319_2026_715_MOESM6_ESM.zip › Figure 4/Figure 4F/hPDl1-WT-cropped-mgfp.jpg]

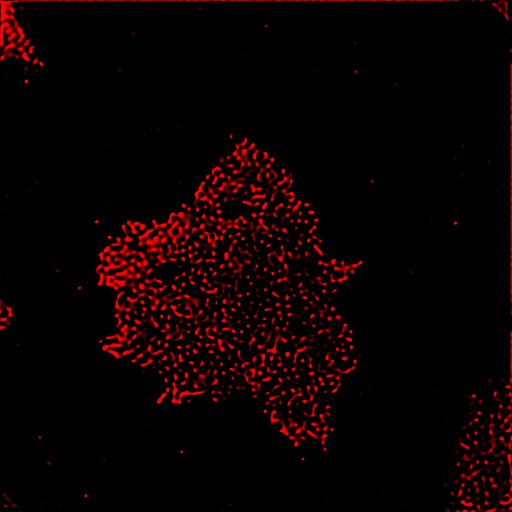

Supplement: Supplementary file 6 — Source data Fig. 4 [file 44319_2026_715_MOESM6_ESM.zip › Figure 4/Figure 4F/hPDl1-WT-mCherry.jpg]

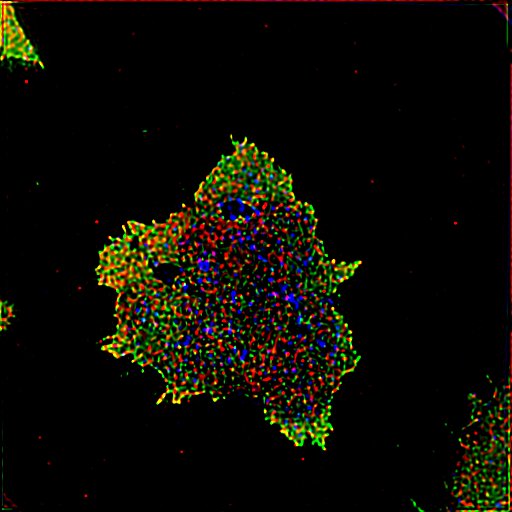

Supplement: Supplementary file 6 — Source data Fig. 4 [file 44319_2026_715_MOESM6_ESM.zip › Figure 4/Figure 4F/hPDl1-WT-merge.jpg]

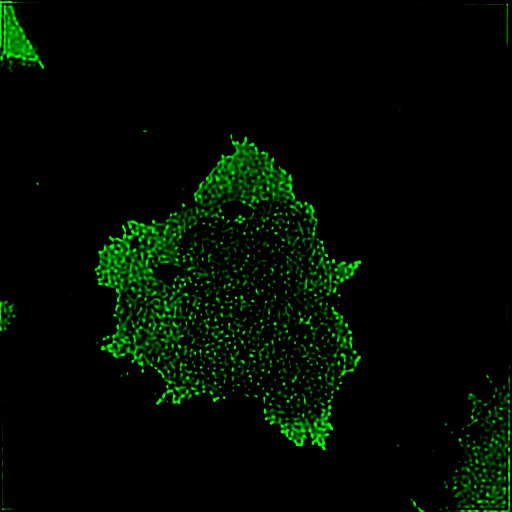

Supplement: Supplementary file 6 — Source data Fig. 4 [file 44319_2026_715_MOESM6_ESM.zip › Figure 4/Figure 4F/hPDl1-WT-mGFP.jpg]

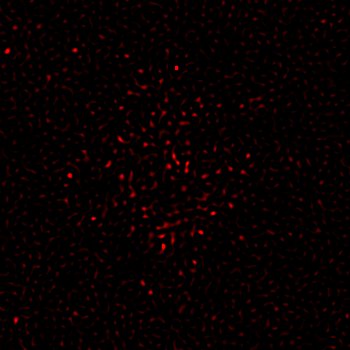

Supplement: Supplementary file 6 — Source data Fig. 4 [file 44319_2026_715_MOESM6_ESM.zip › Figure 4/Figure 4F/I54A-cropped-mcherry.jpg]

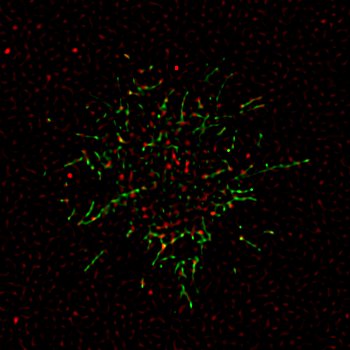

Supplement: Supplementary file 6 — Source data Fig. 4 [file 44319_2026_715_MOESM6_ESM.zip › Figure 4/Figure 4F/I54A-cropped-merge.jpg]

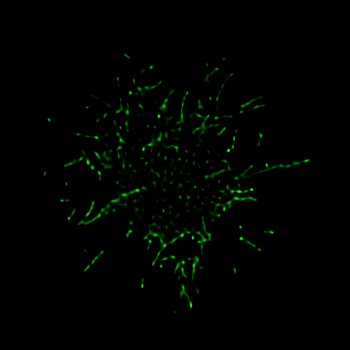

Supplement: Supplementary file 6 — Source data Fig. 4 [file 44319_2026_715_MOESM6_ESM.zip › Figure 4/Figure 4F/I54A-cropped-mgfp.jpg]

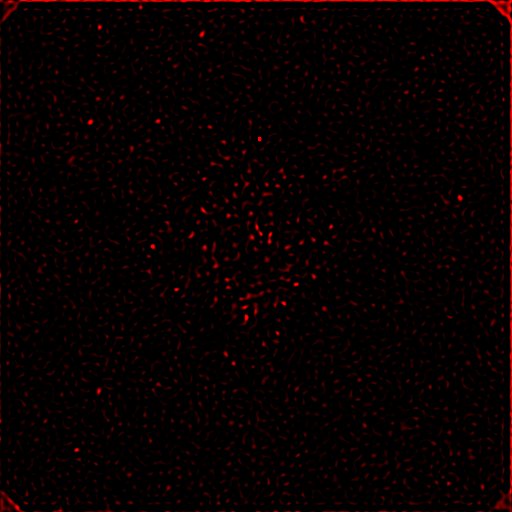

Supplement: Supplementary file 6 — Source data Fig. 4 [file 44319_2026_715_MOESM6_ESM.zip › Figure 4/Figure 4F/I54A-mCherry.jpg]

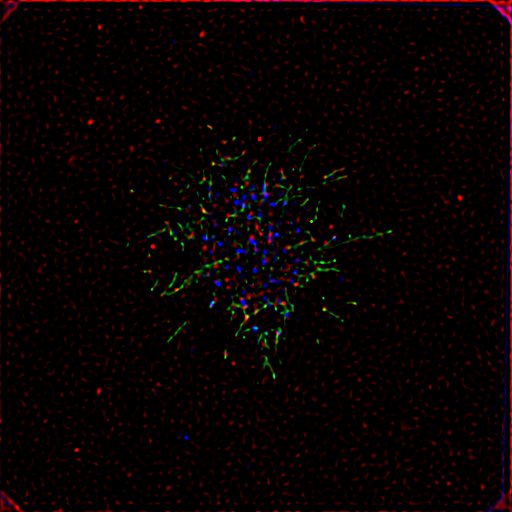

Supplement: Supplementary file 6 — Source data Fig. 4 [file 44319_2026_715_MOESM6_ESM.zip › Figure 4/Figure 4F/I54A-merge.jpg]

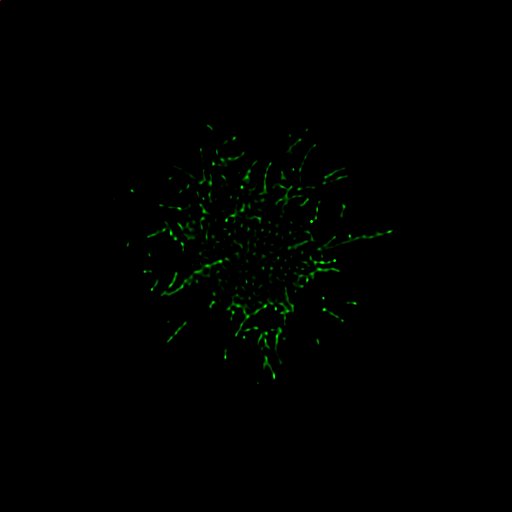

Supplement: Supplementary file 6 — Source data Fig. 4 [file 44319_2026_715_MOESM6_ESM.zip › Figure 4/Figure 4F/I54A-mGFP.jpg]

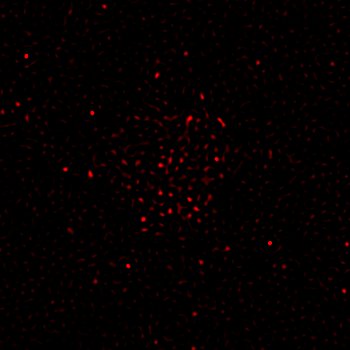

Supplement: Supplementary file 6 — Source data Fig. 4 [file 44319_2026_715_MOESM6_ESM.zip › Figure 4/Figure 4F/K75A-cropped-mcherry.jpg]

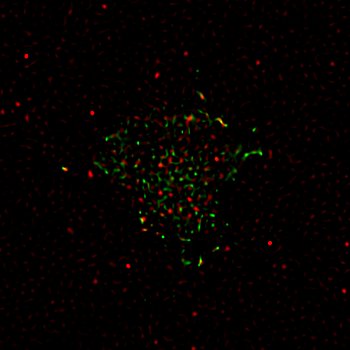

Supplement: Supplementary file 6 — Source data Fig. 4 [file 44319_2026_715_MOESM6_ESM.zip › Figure 4/Figure 4F/K75A-cropped-merge.jpg]

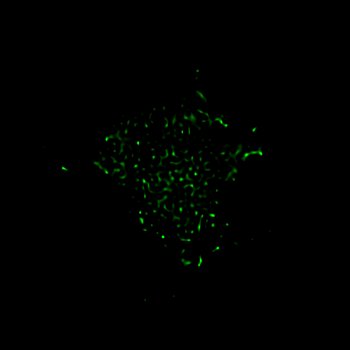

Supplement: Supplementary file 6 — Source data Fig. 4 [file 44319_2026_715_MOESM6_ESM.zip › Figure 4/Figure 4F/K75A-cropped-mgfp.jpg]

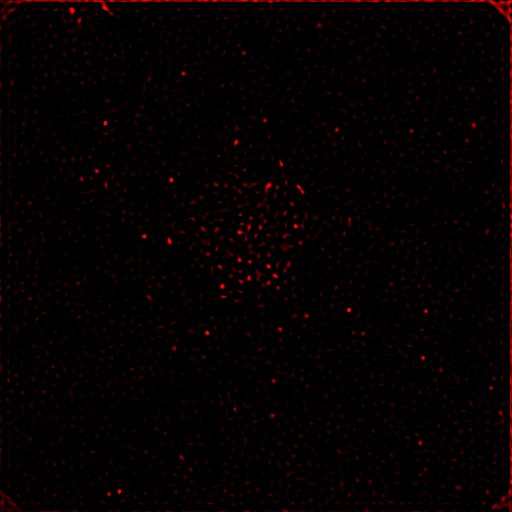

Supplement: Supplementary file 6 — Source data Fig. 4 [file 44319_2026_715_MOESM6_ESM.zip › Figure 4/Figure 4F/K75A-mCherry.jpg]

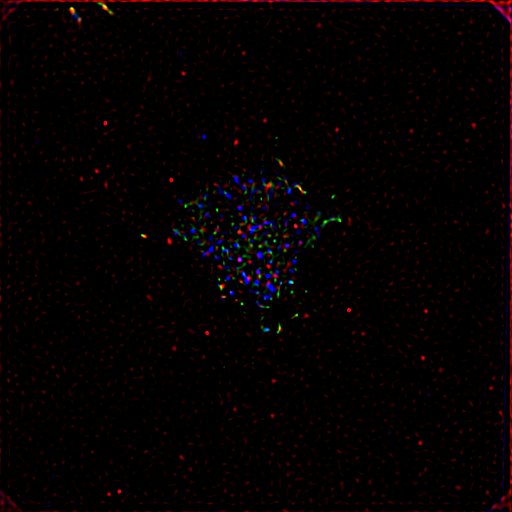

Supplement: Supplementary file 6 — Source data Fig. 4 [file 44319_2026_715_MOESM6_ESM.zip › Figure 4/Figure 4F/K75A-merge.jpg]

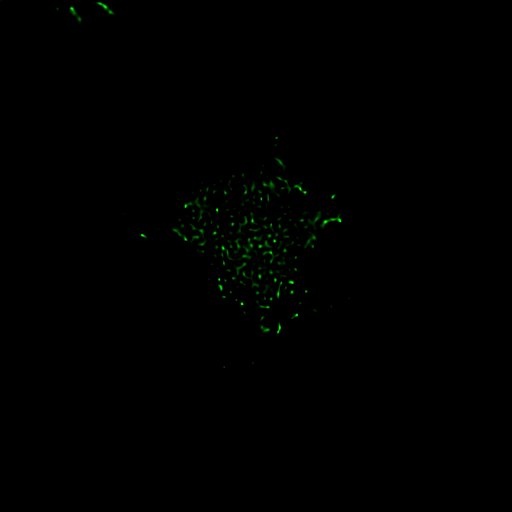

Supplement: Supplementary file 6 — Source data Fig. 4 [file 44319_2026_715_MOESM6_ESM.zip › Figure 4/Figure 4F/K75A-mGFP.jpg]

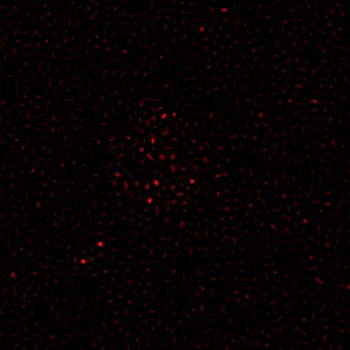

Supplement: Supplementary file 6 — Source data Fig. 4 [file 44319_2026_715_MOESM6_ESM.zip › Figure 4/Figure 4F/Q66A-cropped-mcherry.jpg]

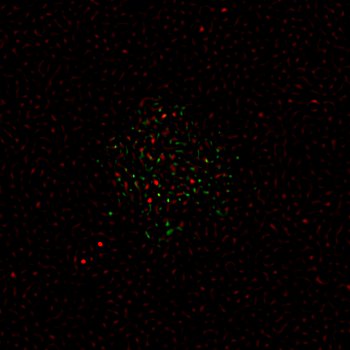

Supplement: Supplementary file 6 — Source data Fig. 4 [file 44319_2026_715_MOESM6_ESM.zip › Figure 4/Figure 4F/Q66A-cropped-merge.jpg]

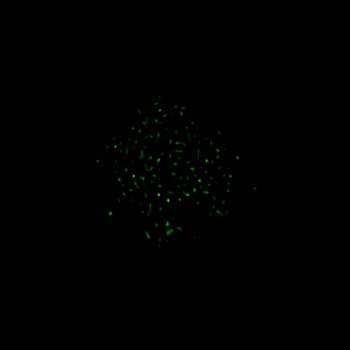

Supplement: Supplementary file 6 — Source data Fig. 4 [file 44319_2026_715_MOESM6_ESM.zip › Figure 4/Figure 4F/Q66A-cropped-mgfp.jpg]

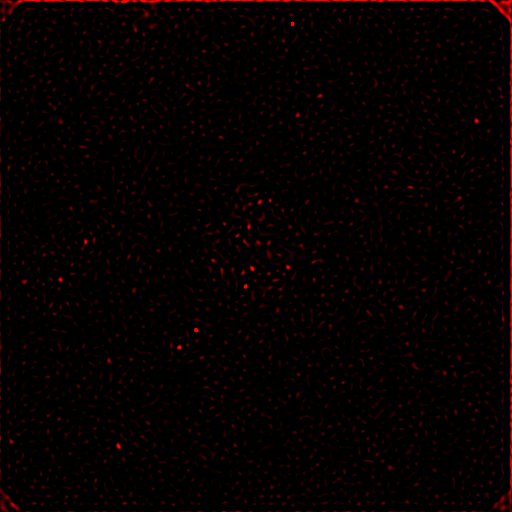

Supplement: Supplementary file 6 — Source data Fig. 4 [file 44319_2026_715_MOESM6_ESM.zip › Figure 4/Figure 4F/Q66A-mCherry.jpg]

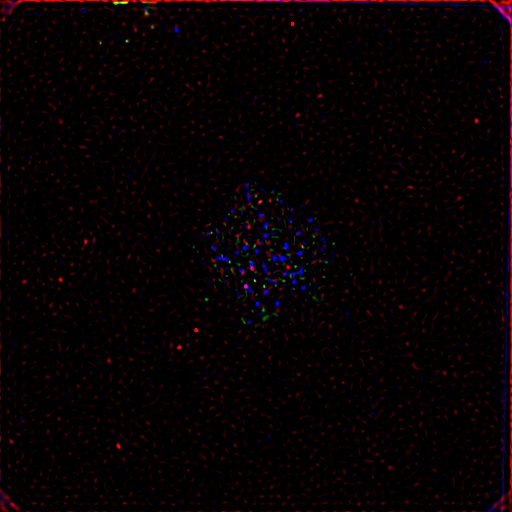

Supplement: Supplementary file 6 — Source data Fig. 4 [file 44319_2026_715_MOESM6_ESM.zip › Figure 4/Figure 4F/Q66A-merge.jpg]

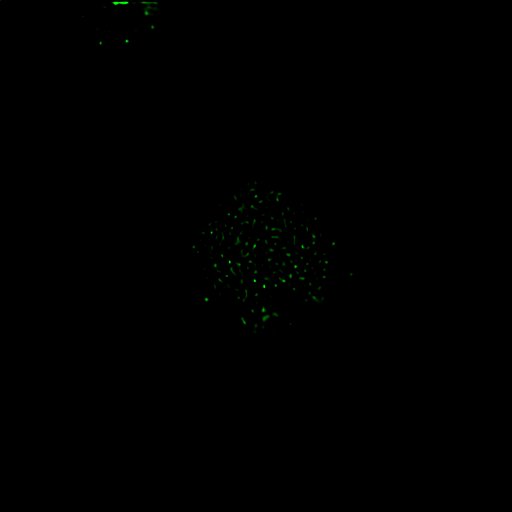

Supplement: Supplementary file 6 — Source data Fig. 4 [file 44319_2026_715_MOESM6_ESM.zip › Figure 4/Figure 4F/Q66A-mGFP.jpg]

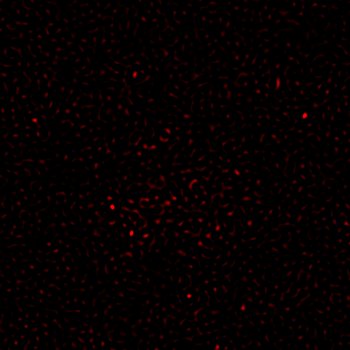

Supplement: Supplementary file 6 — Source data Fig. 4 [file 44319_2026_715_MOESM6_ESM.zip › Figure 4/Figure 4F/R12E-cropped-mcherry.jpg]

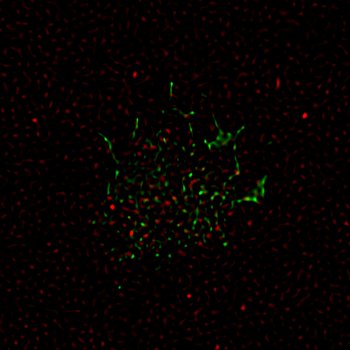

Supplement: Supplementary file 6 — Source data Fig. 4 [file 44319_2026_715_MOESM6_ESM.zip › Figure 4/Figure 4F/R12E-cropped-merge.jpg]

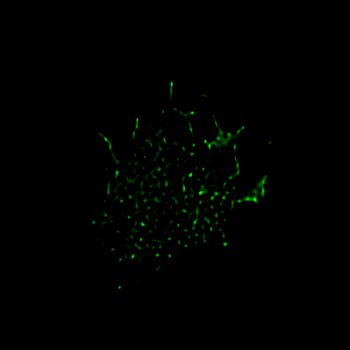

Supplement: Supplementary file 6 — Source data Fig. 4 [file 44319_2026_715_MOESM6_ESM.zip › Figure 4/Figure 4F/R12E-cropped-mgfp.jpg]

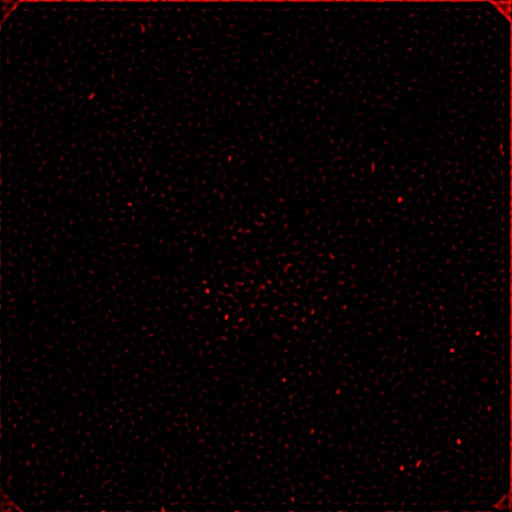

Supplement: Supplementary file 6 — Source data Fig. 4 [file 44319_2026_715_MOESM6_ESM.zip › Figure 4/Figure 4F/R12E-mCherry.jpg]

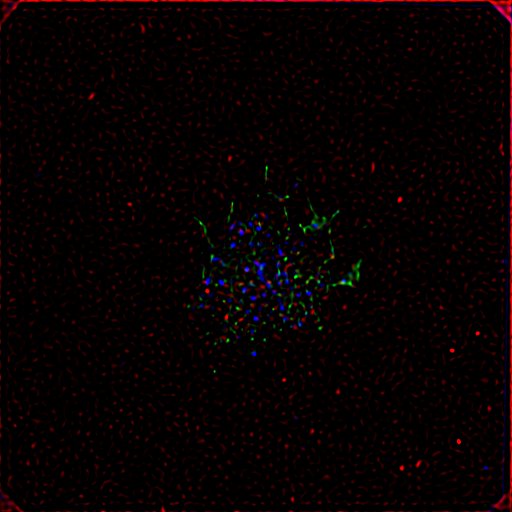

Supplement: Supplementary file 6 — Source data Fig. 4 [file 44319_2026_715_MOESM6_ESM.zip › Figure 4/Figure 4F/R12E-merge.jpg]

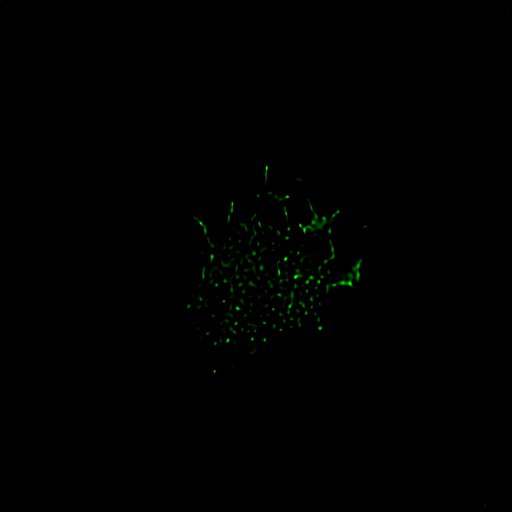

Supplement: Supplementary file 6 — Source data Fig. 4 [file 44319_2026_715_MOESM6_ESM.zip › Figure 4/Figure 4F/R12E-mGFP.jpg]

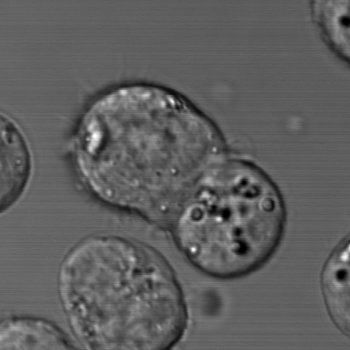

Supplement: Supplementary file 7 — Source data Fig. 5 [file 44319_2026_715_MOESM7_ESM.zip › Figure 5/Figure 5C/Anti-1-DIC.jpg]

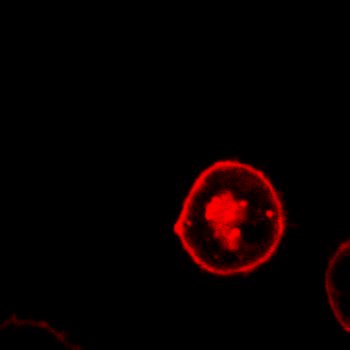

Supplement: Supplementary file 7 — Source data Fig. 5 [file 44319_2026_715_MOESM7_ESM.zip › Figure 5/Figure 5C/Anti-1-mCherry.jpg]

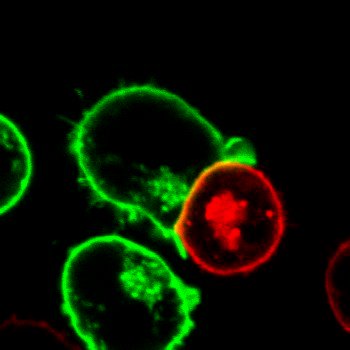

Supplement: Supplementary file 7 — Source data Fig. 5 [file 44319_2026_715_MOESM7_ESM.zip › Figure 5/Figure 5C/Anti-1-Merge.jpg]

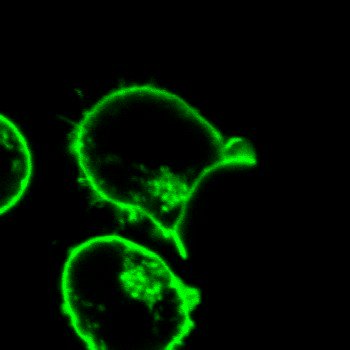

Supplement: Supplementary file 7 — Source data Fig. 5 [file 44319_2026_715_MOESM7_ESM.zip › Figure 5/Figure 5C/Anti-1-mGFP.jpg]

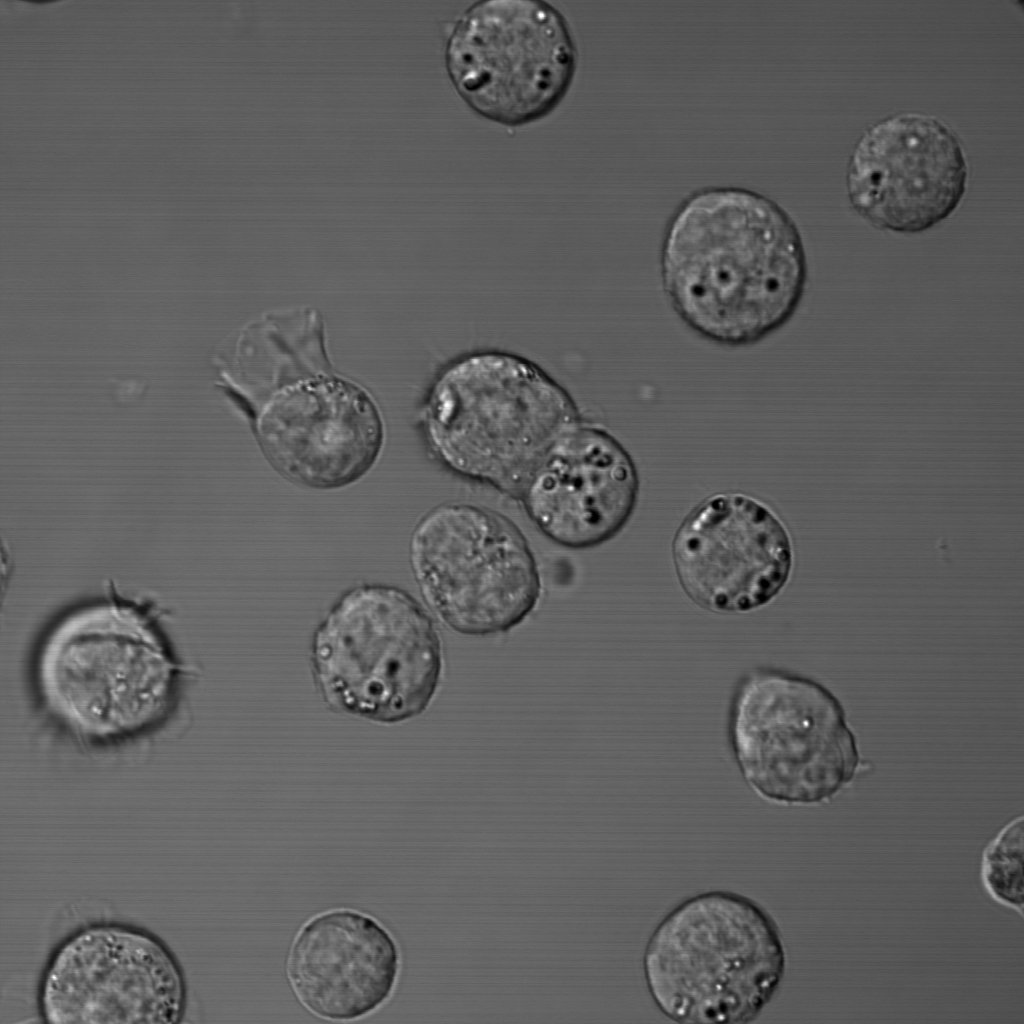

Supplement: Supplementary file 7 — Source data Fig. 5 [file 44319_2026_715_MOESM7_ESM.zip › Figure 5/Figure 5C/Anti-1-uncropped-DIC.jpg]

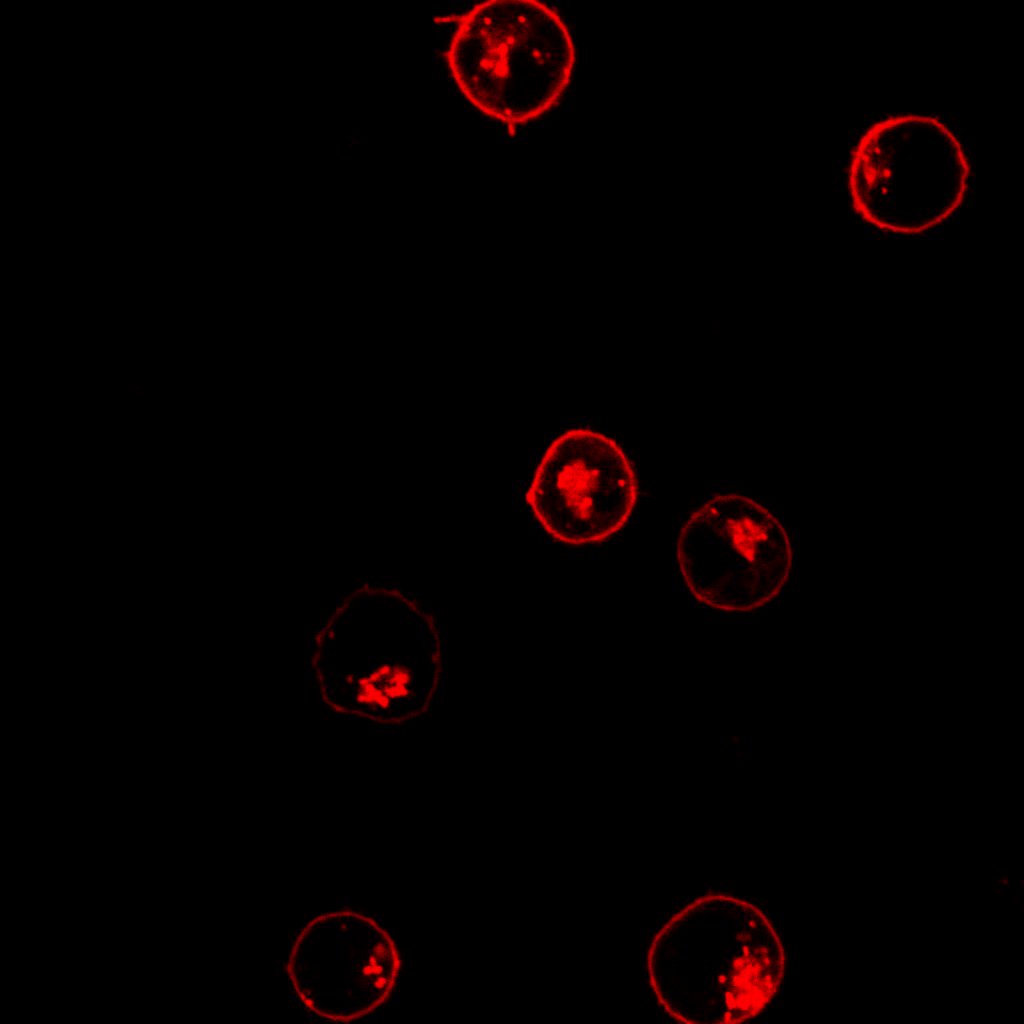

Supplement: Supplementary file 7 — Source data Fig. 5 [file 44319_2026_715_MOESM7_ESM.zip › Figure 5/Figure 5C/Anti-1-uncropped-mCherry.jpg]

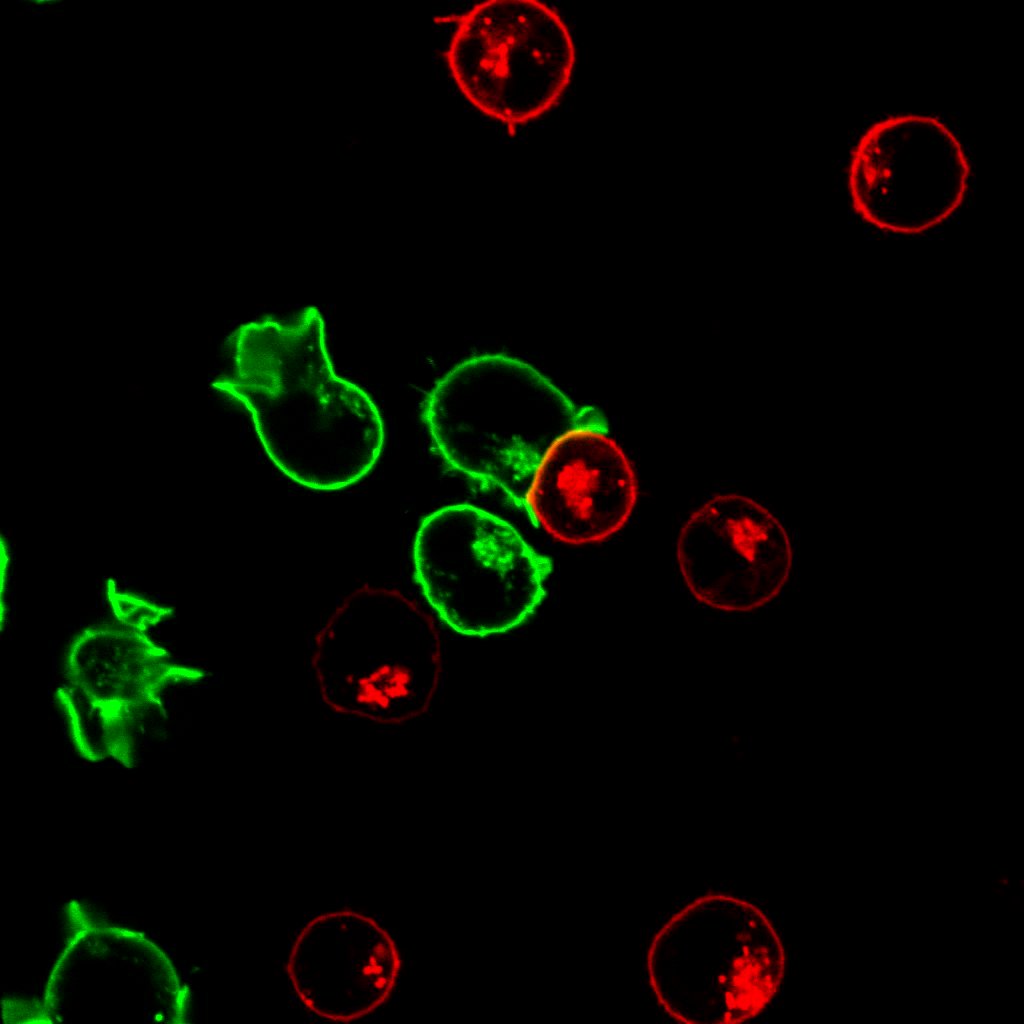

Supplement: Supplementary file 7 — Source data Fig. 5 [file 44319_2026_715_MOESM7_ESM.zip › Figure 5/Figure 5C/Anti-1-uncropped-Merge.jpg]

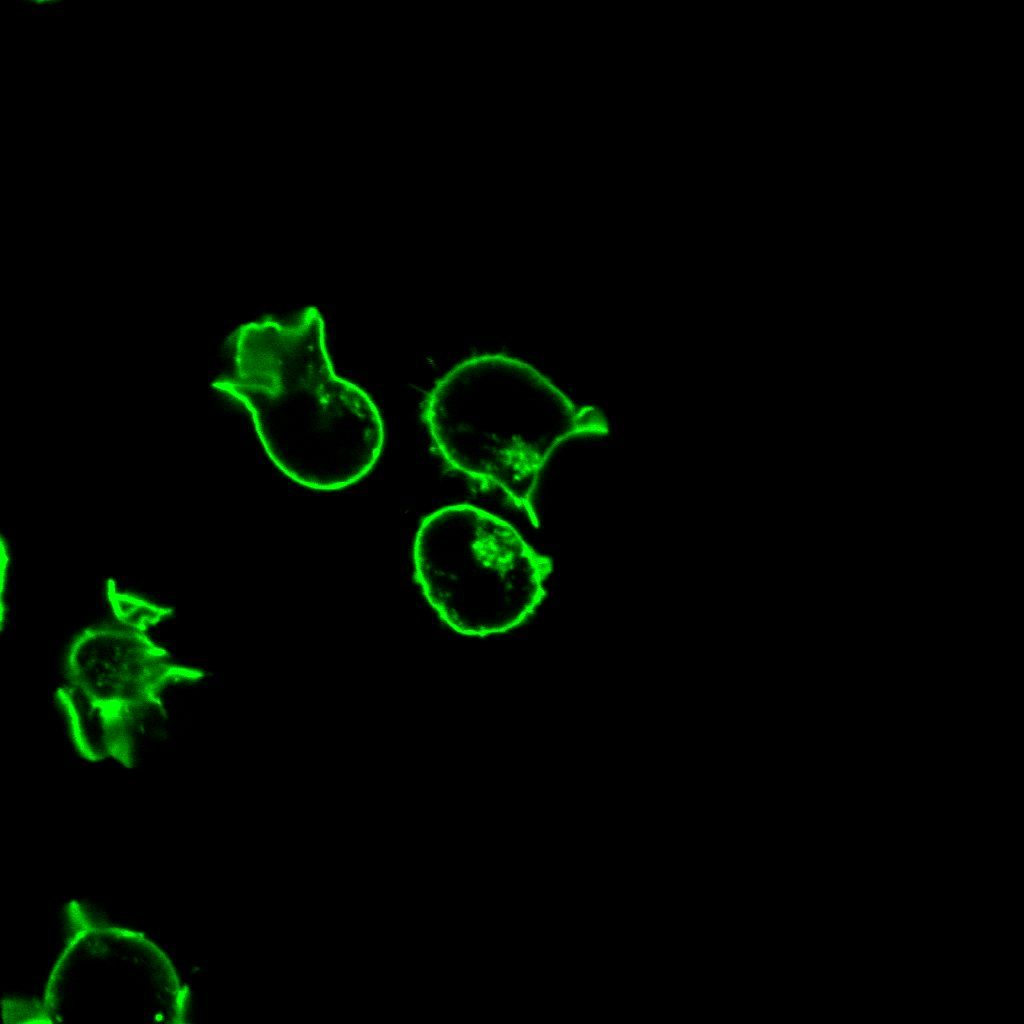

Supplement: Supplementary file 7 — Source data Fig. 5 [file 44319_2026_715_MOESM7_ESM.zip › Figure 5/Figure 5C/Anti-1-uncropped-mGFP.jpg]

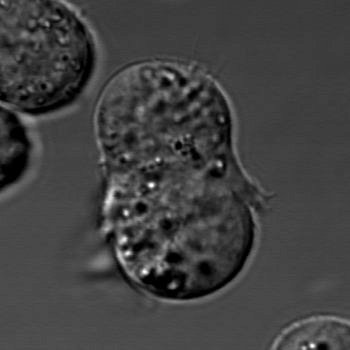

Supplement: Supplementary file 7 — Source data Fig. 5 [file 44319_2026_715_MOESM7_ESM.zip › Figure 5/Figure 5C/L1-1-DIC.jpg]

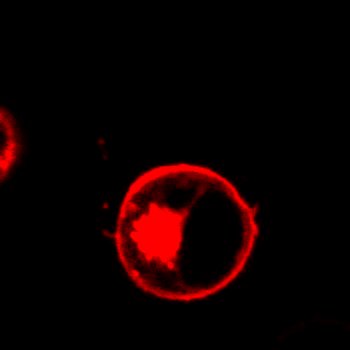

Supplement: Supplementary file 7 — Source data Fig. 5 [file 44319_2026_715_MOESM7_ESM.zip › Figure 5/Figure 5C/L1-1-mCherry.jpg]

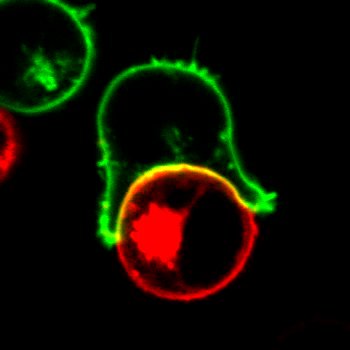

Supplement: Supplementary file 7 — Source data Fig. 5 [file 44319_2026_715_MOESM7_ESM.zip › Figure 5/Figure 5C/L1-1-Merge.jpg]

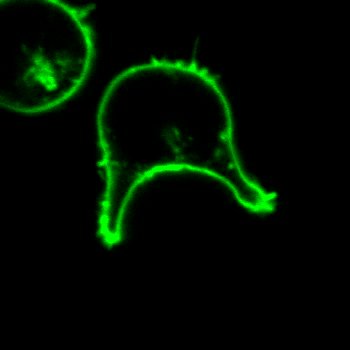

Supplement: Supplementary file 7 — Source data Fig. 5 [file 44319_2026_715_MOESM7_ESM.zip › Figure 5/Figure 5C/L1-1-mGFP.jpg]

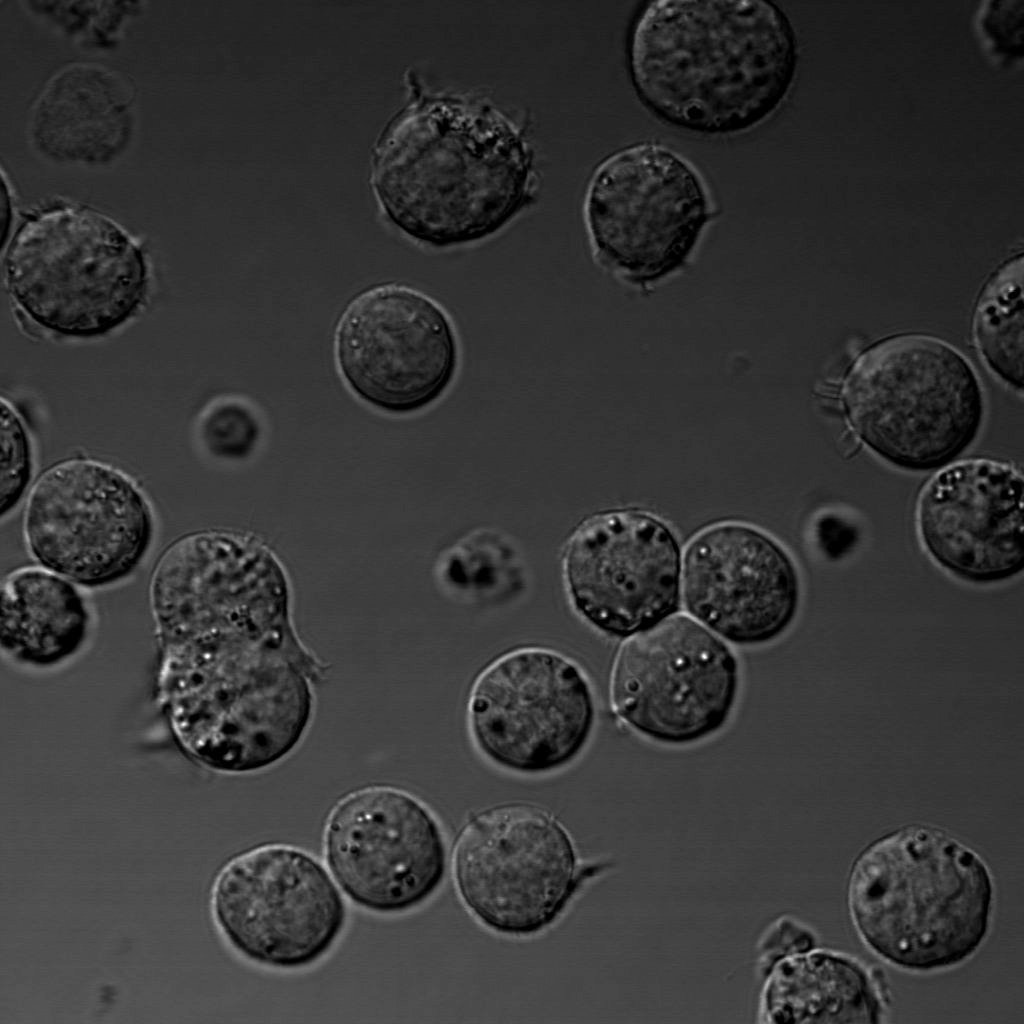

Supplement: Supplementary file 7 — Source data Fig. 5 [file 44319_2026_715_MOESM7_ESM.zip › Figure 5/Figure 5C/L1-1-uncropped-DIC.jpg]

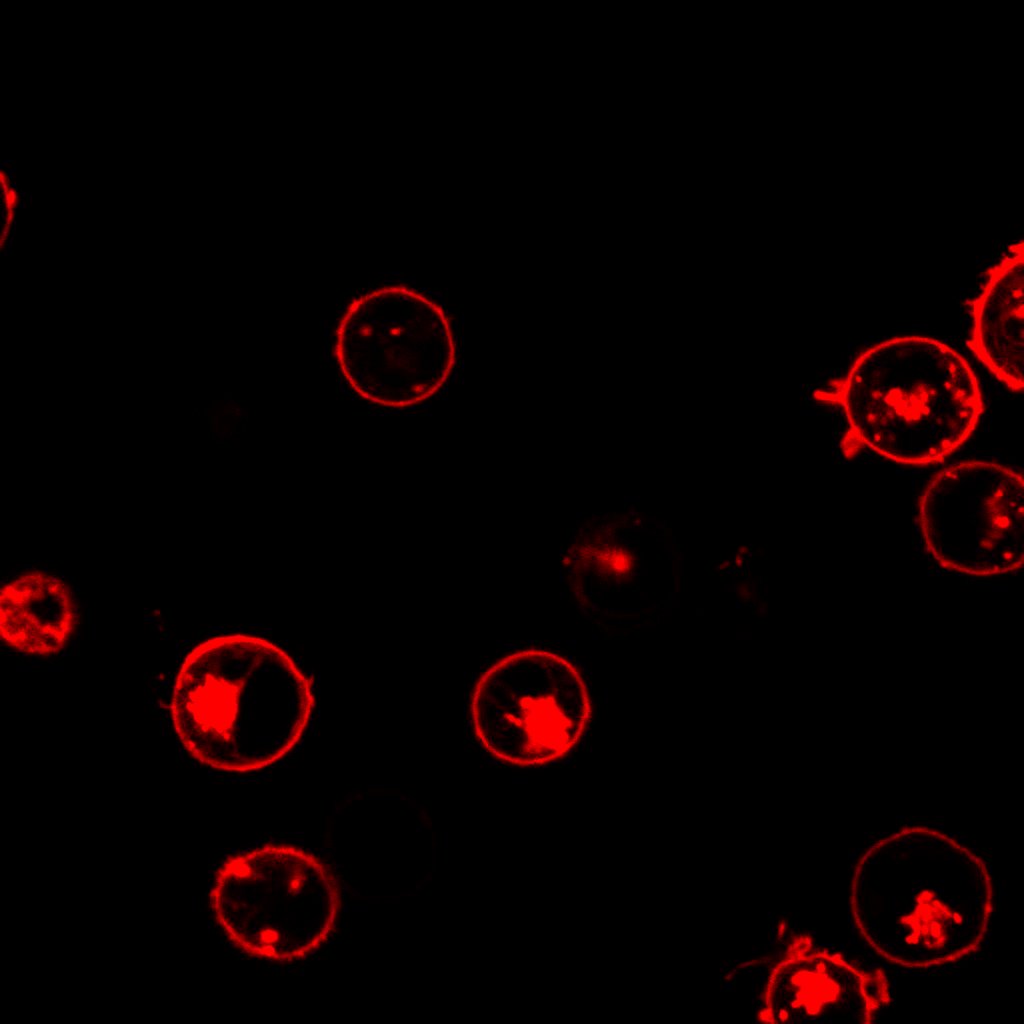

Supplement: Supplementary file 7 — Source data Fig. 5 [file 44319_2026_715_MOESM7_ESM.zip › Figure 5/Figure 5C/L1-1-uncropped-mCherry.jpg]

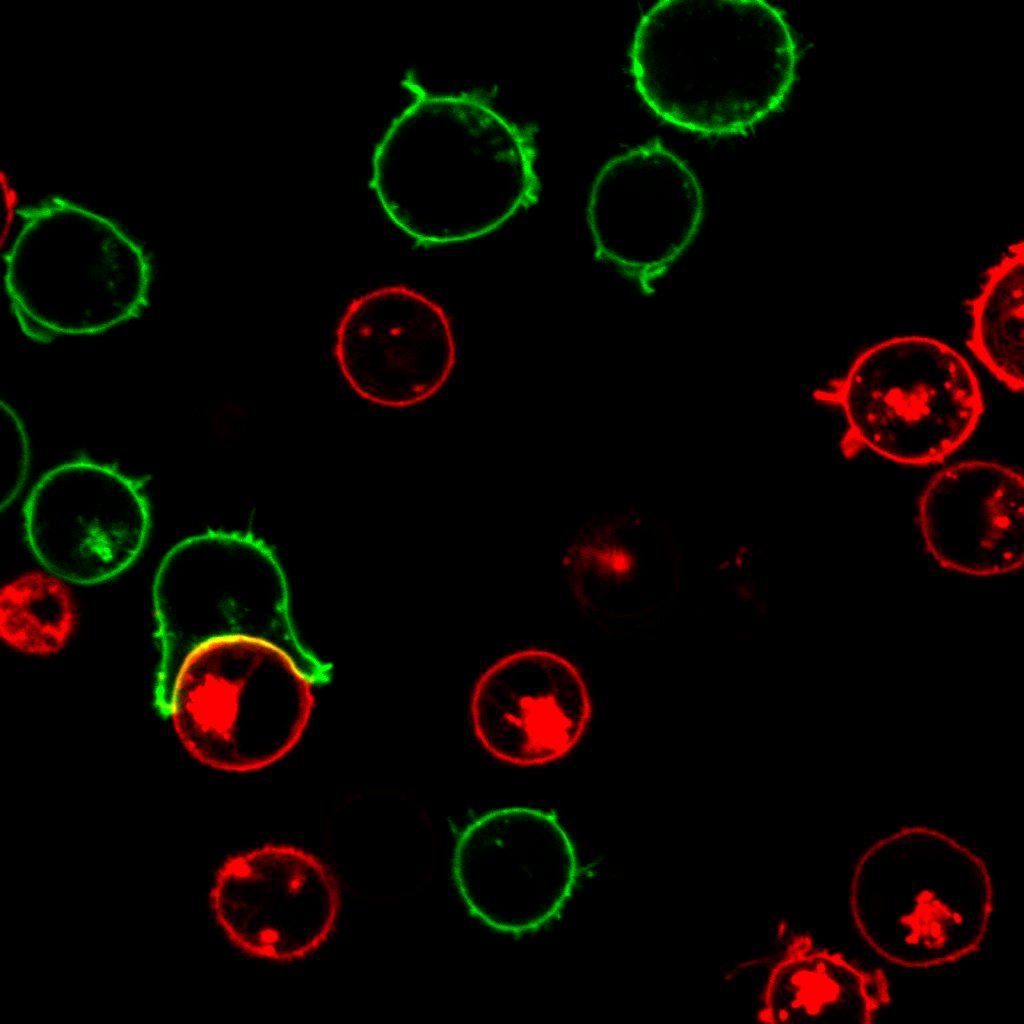

Supplement: Supplementary file 7 — Source data Fig. 5 [file 44319_2026_715_MOESM7_ESM.zip › Figure 5/Figure 5C/L1-1-uncropped-Merge.jpg]

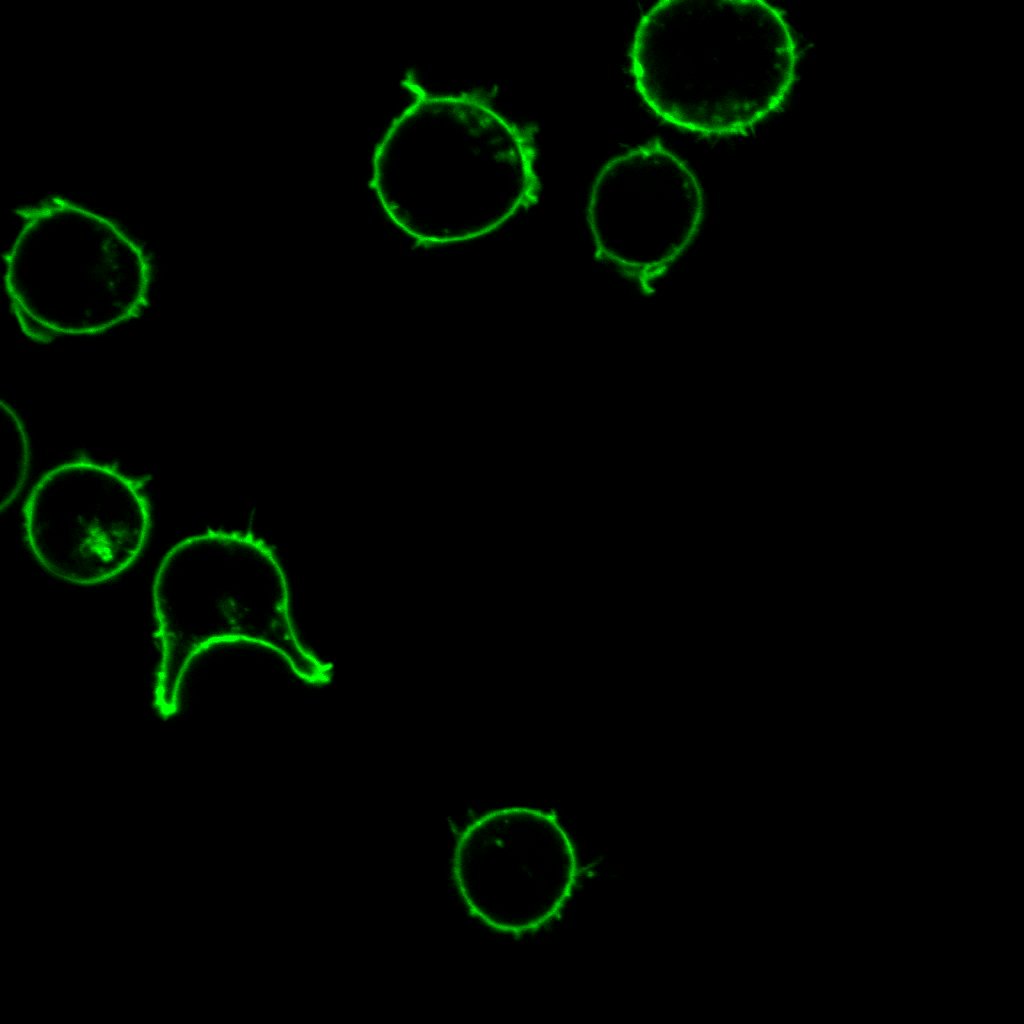

Supplement: Supplementary file 7 — Source data Fig. 5 [file 44319_2026_715_MOESM7_ESM.zip › Figure 5/Figure 5C/L1-1-uncropped-mGFP.jpg]

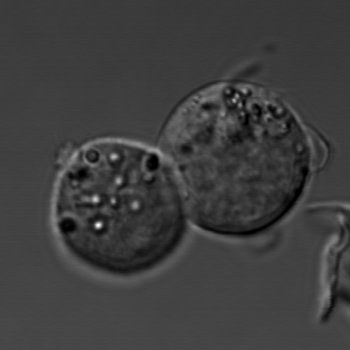

Supplement: Supplementary file 7 — Source data Fig. 5 [file 44319_2026_715_MOESM7_ESM.zip › Figure 5/Figure 5C/Mock-1-DIC.jpg]

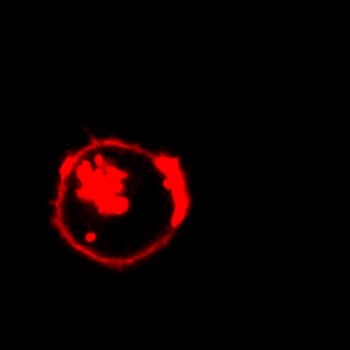

Supplement: Supplementary file 7 — Source data Fig. 5 [file 44319_2026_715_MOESM7_ESM.zip › Figure 5/Figure 5C/Mock-1-mCherry.jpg]

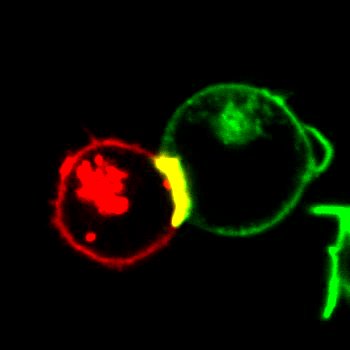

Supplement: Supplementary file 7 — Source data Fig. 5 [file 44319_2026_715_MOESM7_ESM.zip › Figure 5/Figure 5C/Mock-1-Merge.jpg]

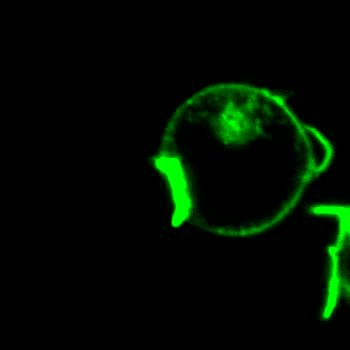

Supplement: Supplementary file 7 — Source data Fig. 5 [file 44319_2026_715_MOESM7_ESM.zip › Figure 5/Figure 5C/Mock-1-mGFP.jpg]

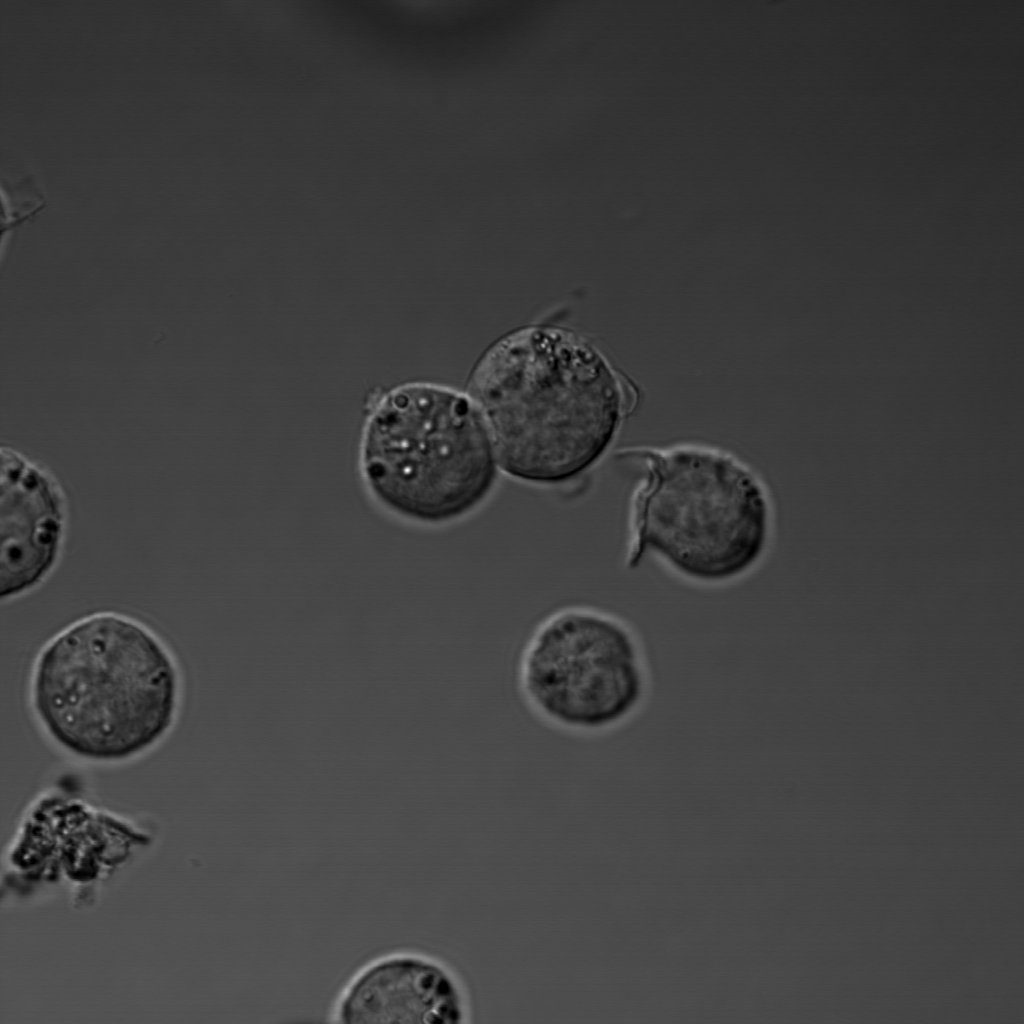

Supplement: Supplementary file 7 — Source data Fig. 5 [file 44319_2026_715_MOESM7_ESM.zip › Figure 5/Figure 5C/Mock-1-uncropped-DIC.jpg]

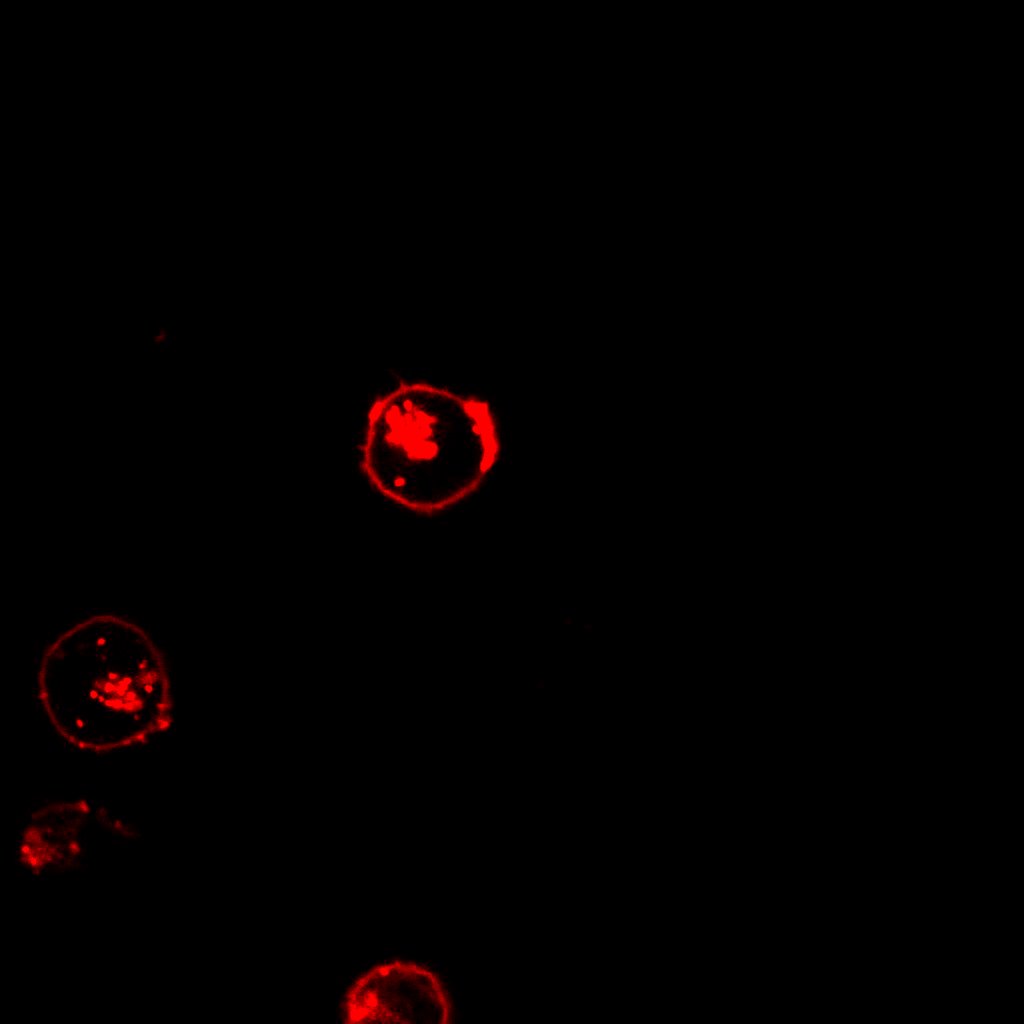

Supplement: Supplementary file 7 — Source data Fig. 5 [file 44319_2026_715_MOESM7_ESM.zip › Figure 5/Figure 5C/Mock-1-uncropped-mCherry.jpg]

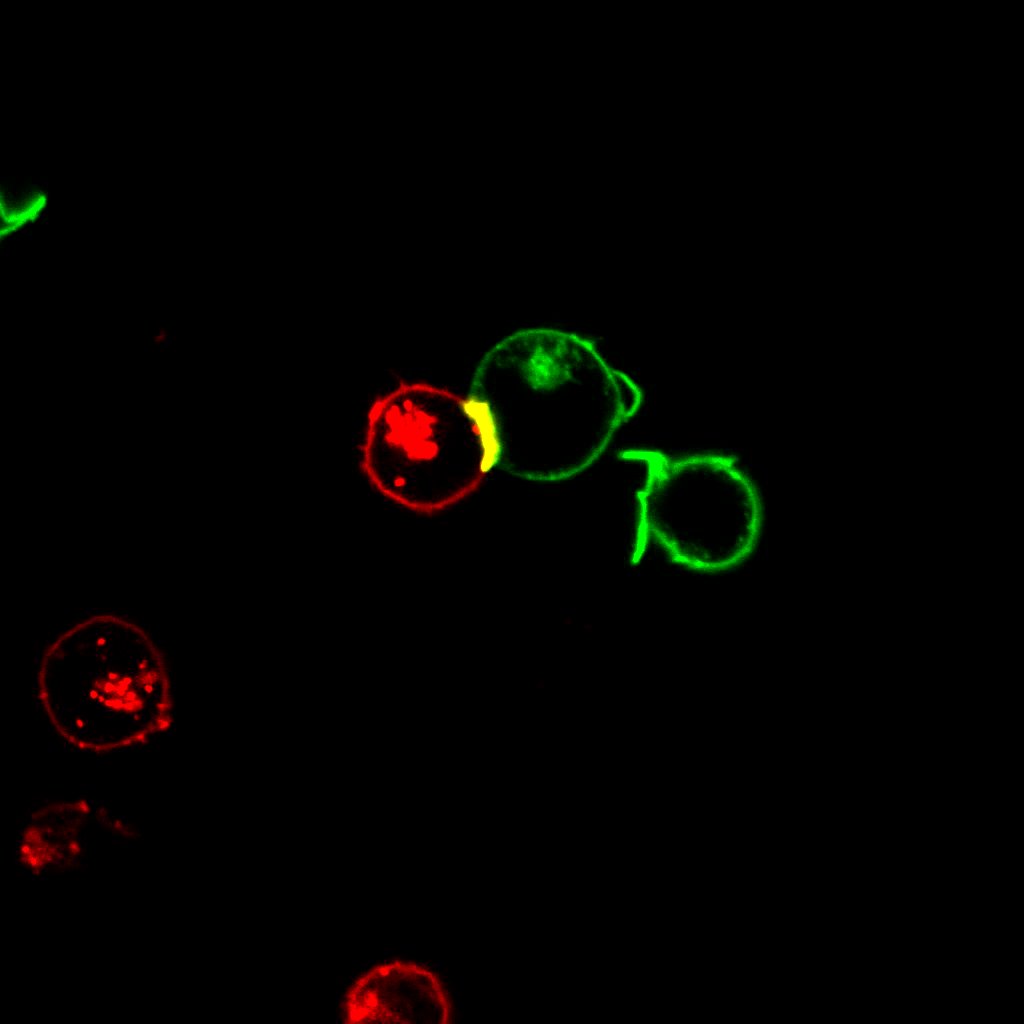

Supplement: Supplementary file 7 — Source data Fig. 5 [file 44319_2026_715_MOESM7_ESM.zip › Figure 5/Figure 5C/Mock-1-uncropped-Merge.jpg]

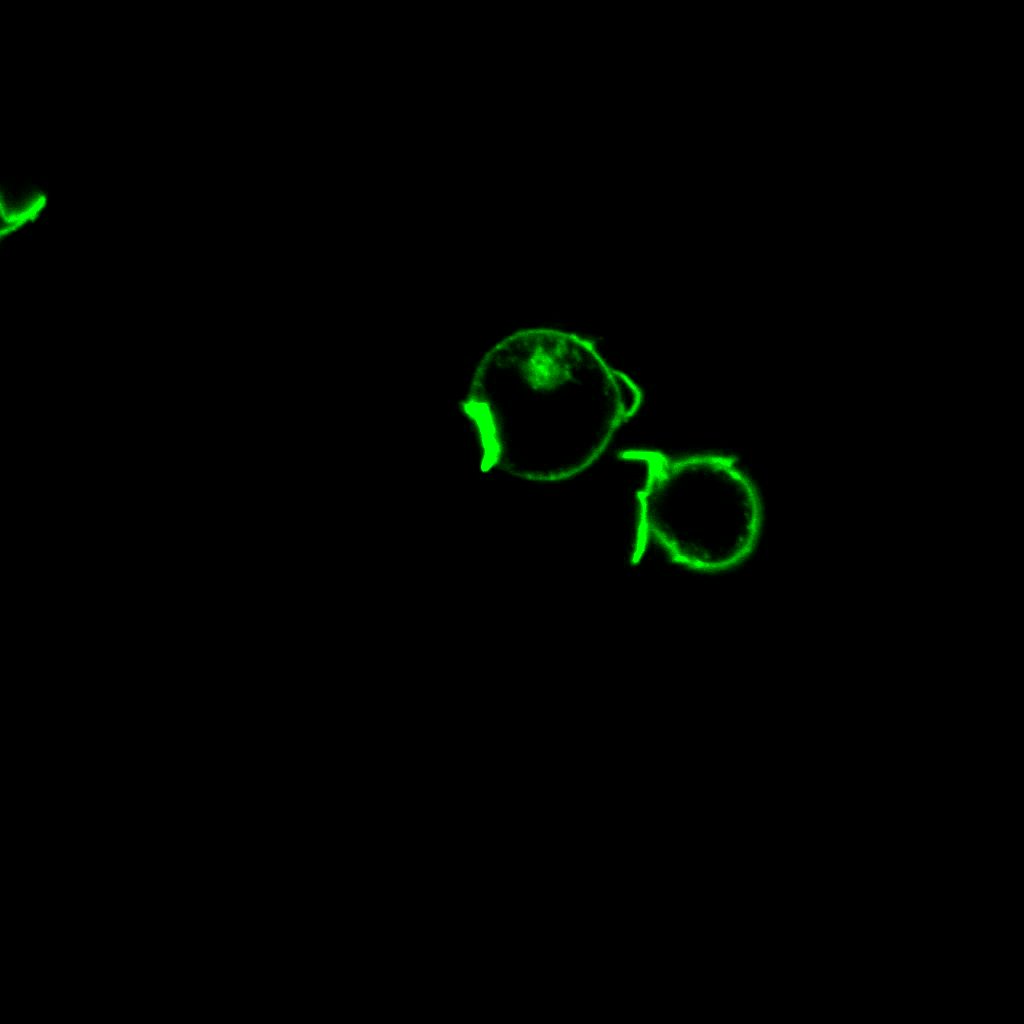

Supplement: Supplementary file 7 — Source data Fig. 5 [file 44319_2026_715_MOESM7_ESM.zip › Figure 5/Figure 5C/Mock-1-uncropped-mGFP.jpg]
