# Supplementary material for: Mechanical force regulates the inhibitory function of PD-1
Source: EMBO Rep. 2026 Feb 25;27(7):1789–812. doi: 10.1038/s44319-026-00715-6 (PMC13076993; doi:10.1038/s44319-026-00715-6)
Supplement: Supplementary file 9 — Expanded View Figures [file 44319_2026_715_MOESM9_ESM.pdf]

Expanded View Figures

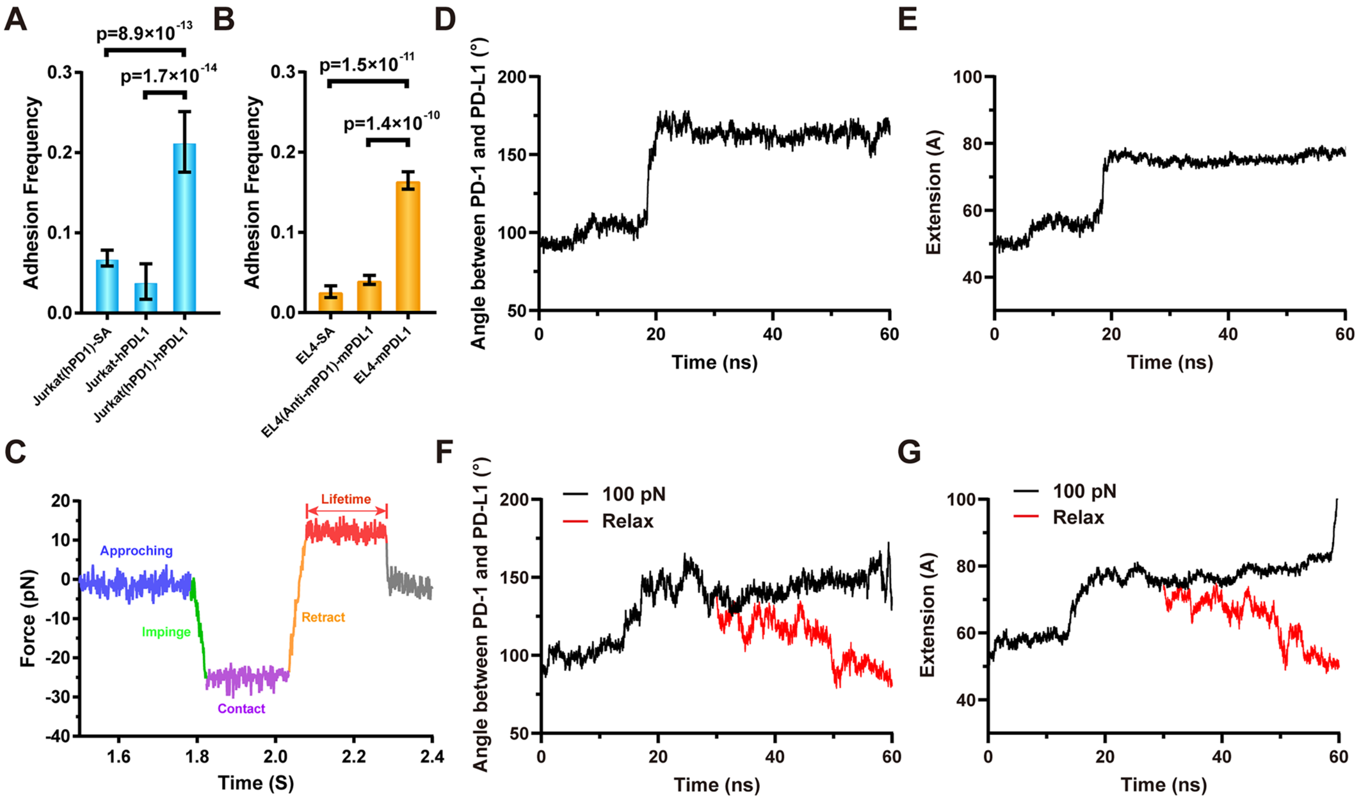

Figure EV1. Characterization of PD1/ligand interactions.

(**A**, **B**) The adhesion frequency of human (**A**) and mouse (**B**) PD-1/PD-L1 interactions, compared to the indicated control, data were shown as mean ± SD, $n = 10$ (**A**), $n = 9$ (**B**); (**C**) Typical force-clamp curve from BFP experiments, different phases were shown in differed colors and indicated; (**D**, **E**) Representative time-course of the inter-domain angle (**D**) and CT-CT distance (**E**) between PD-1 and PD-L2 in cf-SMD simulations; (**F**, **G**). Representative time-course of the inter-domain angle (**F**) and CT-CT distance (**G**) between PD-1 and PD-L2 in cf-SMD simulations (black) and relax simulations (red). Data information: one-way ANOVA is used to produce *p* value in Fig. EV1A, B.

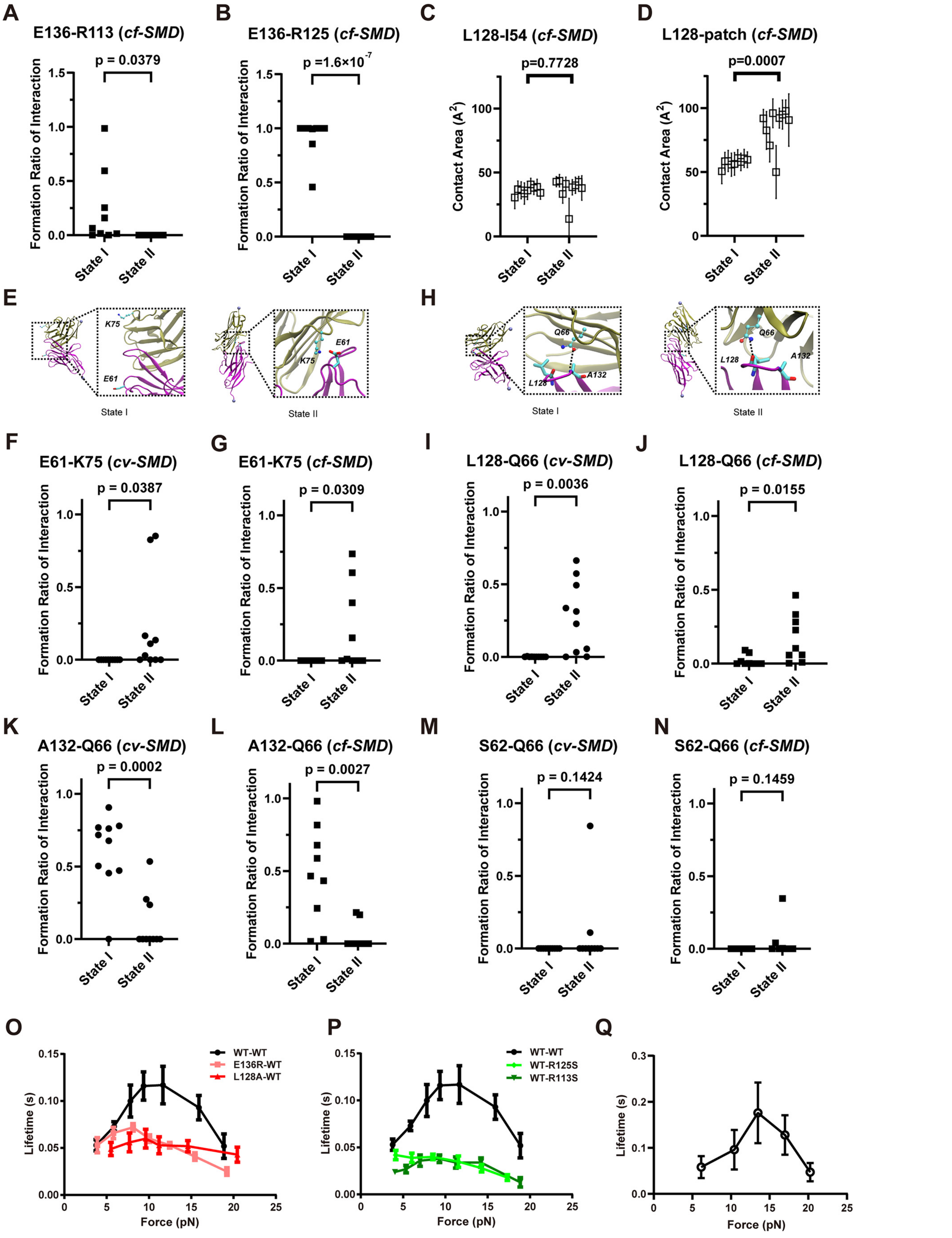
A
E136-R113 (cf-SMD)
p = 0.0379
Formation Ratio of Interaction
State I
State II
B
E136-R125 (cf-SMD)
p =1.6×10-7
C
L128-I54 (cf-SMD)
p=0.7728
Contact Area (A2)
D
L128-patch (cf-SMD)
p=0.0007
E
K75
E61
State I
State II
H
Q66
L128
A132
F
E61-K75 (cv-SMD)
p = 0.0387
G
E61-K75 (cf-SMD)
p = 0.0309
I
L128-Q66 (cv-SMD)
p = 0.0036
J
L128-Q66 (cf-SMD)
p = 0.0155
K
A132-Q66 (cv-SMD)
p = 0.0002
L
A132-Q66 (cf-SMD)
p = 0.0027
M
S62-Q66 (cv-SMD)
p = 0.1424
N
S62-Q66 (cf-SMD)
p = 0.1459
O
WT-WT
E136R-WT
L128A-WT
Lifetime (s)
Force (pN)
P
WT-WT
WT-R125S
WT-R113S
Q

 © The Author(s)

Figure EV2. Key residue pairs identified from MD simulations.

(**A**, **B**) Probabilities of the formation of E136/R113 (**A**) and E136/R125 (**B**) from PD-1 and PD-L1 in cf-SMD simulations ($n = 9$); (**C**, **D**) Analysis of contact area between L128 (PD-1) and I54 (**C**) or hydrophobic patch (**D**) of PD-L1 in cf-SMD simulations ($n = 9$); (**E**) Representative snapshots showing the interaction between E61 (PD-1) and K75 (PD-L1) in the two different binding states; (**F**, **G**) Probabilities of salt bridge formation between E61 (PD-1) and K75 (PD-L1) in cv-SMD simulations (**F**), $n = 10$) and cf-SMD simulations (**G**), $n = 9$); (**H**) Representative snapshots depicting interaction between L128/A132 backbone (PD1) and Q66 sidechain (PD-L1) in the two different binding states; (**I**, **J**) Probabilities of hydrogen bond formation between L128 backbone and Q66 sidechain in cv-SMD simulations (**I**), $n = 10$) and cf-SMD simulations (**J**), $n = 9$); (**K**, **L**) Probabilities of hydrogen bond formation between A132 backbone and Q66 sidechain in cv-SMD simulations (**K**), $n = 10$) and cf-SMD simulations (**L**), $n = 9$); (**M**, **N**). Probabilities of salt bridge formation of S62/Q66 in cv-SMD simulations (**M**), $n = 10$) and cf-SMD simulations (**N**), $n = 9$); (**O**) Mean bond lifetime dependence on force for WT or mutated (as indicated) human PD-1 interacting with WT human PD-L1 (purified from 293F), data were shown as mean ± SEM; (**P**) Mean bond lifetime dependence on force for WT PD-1 interacting with WT or mutated (as indicated) PD-L1 (purified from 293F), data were shown as mean ± SEM. (**Q**) Mean bond lifetime dependence on force for WT PD-1 interacting with WT PD-L1 (both PD-1 and PD-L1 were purified from 293F), data were shown as mean ± SEM.

© The Author(s)

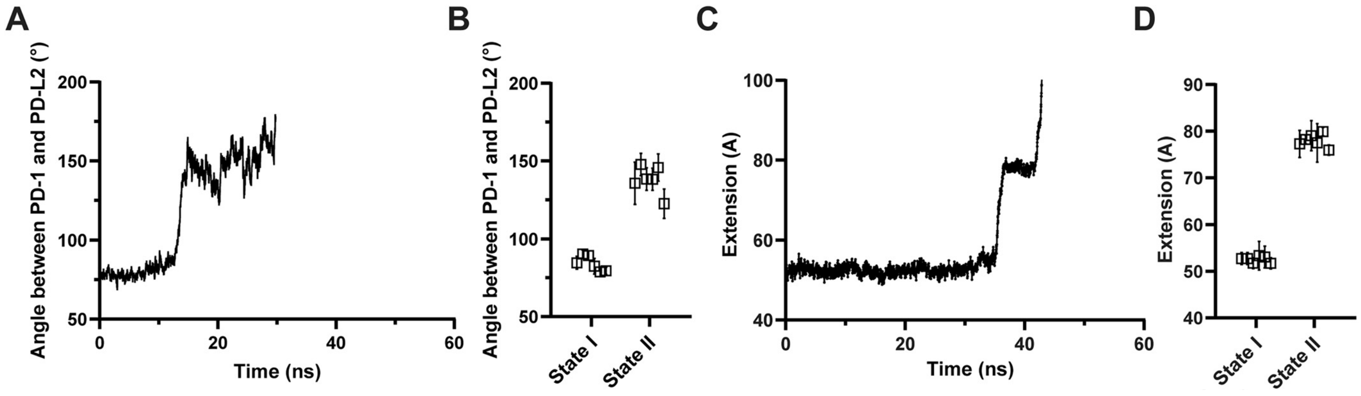

Figure EV3. PD-1/PD-L2 dissociation by cf-SMD simulations.

(**A**) Time-course of the inter-domain angle between PD1 and PD-L2 in one representative cf-SMD simulations, exhibiting two different bending sates; (**B**) Statistics of the inter-domain angle between PD-1 and PD-L2 for the two binding states in cf-SMD simulations ($n = 6$); (**C**) Time-course of the CT-CT distance between PD-1 and PD-L2 in the representative cf-SMD simulation shown in (**A**); (**D**) Statistics of the CT-CT distance between PD-1 and PD-L2 for the two different binding states in cf-SMD simulations ($n = 6$).

 © The Author(s)

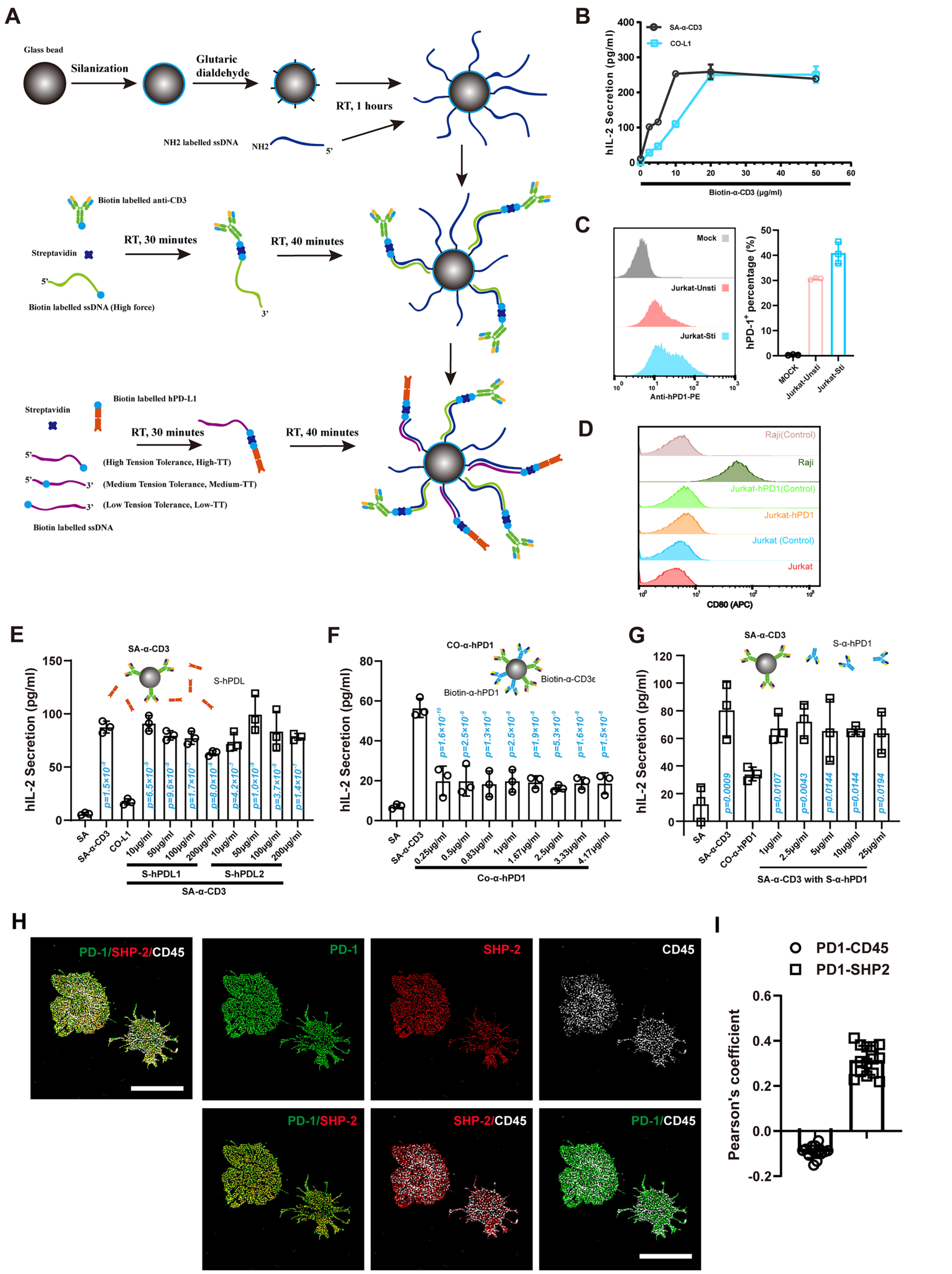
A
Glass bead
Silanization
Glutaric dialdehyde
RT, 1 hours
NH2 labelled ssDNA
Biotin labelled anti-CD3
Streptavidin
RT, 30 minutes
RT, 40 minutes
Biotin labelled ssDNA (High force)
Biotin labelled hPD-L1
(High Tension Tolerance, High-TT)
(Medium Tension Tolerance, Medium-TT)
(Low Tension Tolerance, Low-TT)
Biotin labelled ssDNA
B
SA-α-CD3
CO-L1
hIL-2 Secretion (pg/ml)
Biotin-α-CD3 (μg/ml)
C
Mock
Jurkat-Unsti
Jurkat-Sti
Anti-hPD1-PE
hPD-1+ percentage (%)
D
Raji(Control)
Raji
Jurkat-hPD1(Control)
Jurkat-hPD1
Jurkat (Control)
Jurkat
CD80 (APC)
E
SA-α-CD3
S-hPDL
S-hPDL1
S-hPDL2
F
CO-α-hPD1
Biotin-α-hPD1
Biotin-α-CD3ε
Co-α-hPD1
G
SA-α-CD3
S-α-hPD1
SA-α-CD3 with S-α-hPD1
H
PD-1/SHP-2/CD45
PD-1
SHP-2
CD45
PD-1/SHP-2
SHP-2/CD45
PD-1/CD45
I
PD1-CD45
PD1-SHP2
Pearson's coefficient

© The Author(s)

Figure EV4. Ligand immobilization is essential for PD-1-mediated T cell inhibition.

(**A**) Fabrication of DNA-based tension gauge tether on glass beads (details see Methods); (**B**) IL-2 secretion of Jurkat cells cocultured with or without PD-L1 (15 μg/ml), data were shown as mean ± SEM, $n = 3$; (**C**) Cell surface expression of PD-1 in stimulated and unstimulated Jurkat cells, data are shown as mean ± SD, $n = 3$; (**D**) The expression of CD80 in two Jurkat cell lines (Jurkat-wt and human PD-1 overexpressed Jurkat cell) and Raji cell; (**E–G**) IL-2 secretion of Jurkat cells stimulated with SA-α-CD3 beads in the presence of soluble PD-L1/PD-L2 (**E**), CO-α-hPD1 beads alone (**F**), or SA-α-CD3 beads with soluble PD-L1 antibody (**G**), data were shown as mean ± SD, $n = 3$; (**H**, **I**). The distribution of CD45 and SHP2 in PD-L1 stimulated Jurkat cells (**H**), the Pearson's coefficient between PD1/CD45 and PD1/SHP2 were quantified (**I**). Scale bar: 20 μm, $n = 14$. Data information: In panels (**B**, **E–G**), 2.5 μg/ml soluble anti-CD28 was used in the experiments, and all the experiments were technically repeated three times. One-way ANOVA is used to produce p value in Fig. EV4D–F.

© The Author(s)

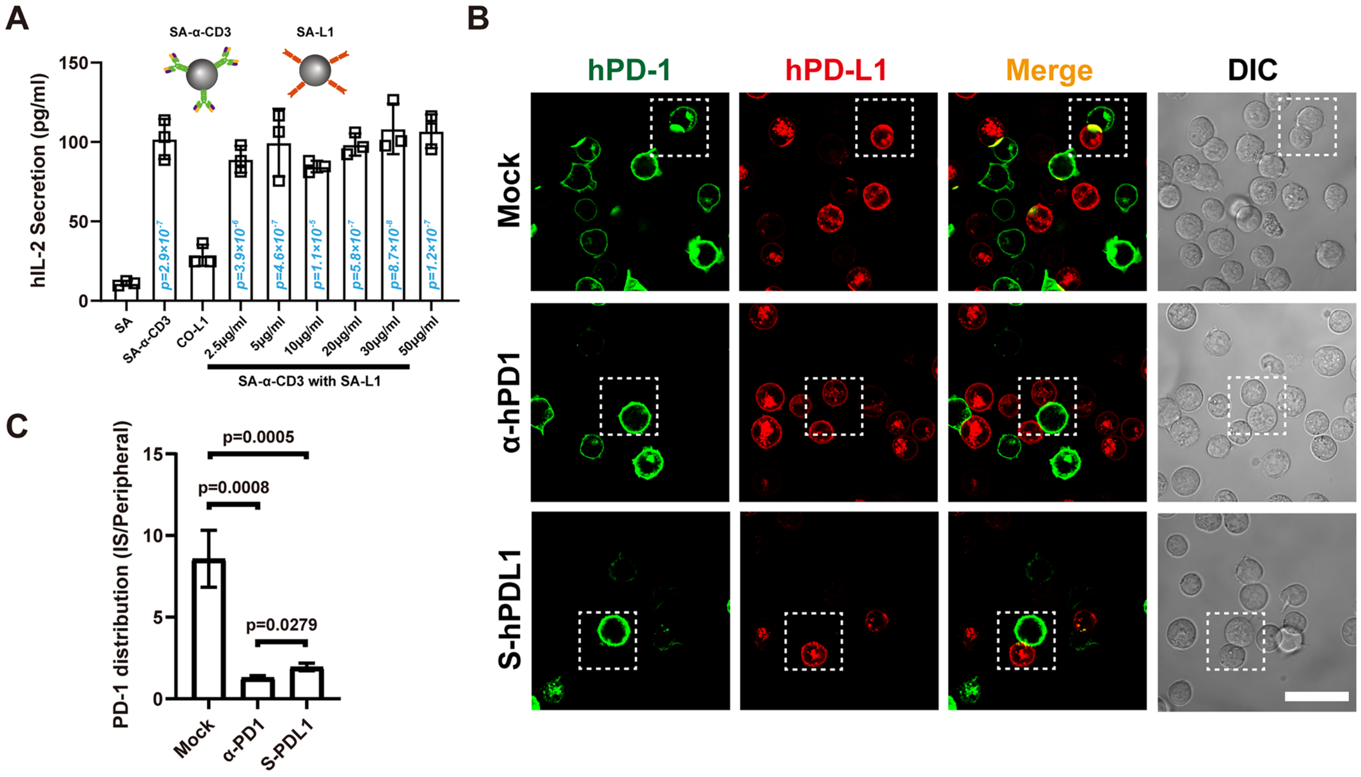

Figure EV5. PD-1-mediated inhibitory function depends on its IS localization.

(**A**) IL-2 secretion of Jurkat cells stimulated with anti-CD3 in the presence of co-immobilized PD-L1 (CO-L1) or PD-L1 immobilized on separated beads with indicated coating concentration, data were shown as mean ± SD, $n = 3$; (**B**) Representative confocal image of Jurkat (hPD1$^+$)-Raji (WT or hPD-L1$^+$) conjugates. Cells were imaged to visualize the interaction between Jurkat cells expressing human PD-1 (green) and Raji cells expressing PD-L1 (red); (**C**) Quantification of PD-1 engagement on cell–cell conjugates. Data were shown as mean ± SEM, Mock: $n = 30$, αPD-1: $n = 22$, S-PDL1: $n = 29$. Data information: One-way ANOVA is used to produce p value in Fig. EV5A, t-test is used to quantify the difference between groups in Fig. EV5C.

© The Author(s)

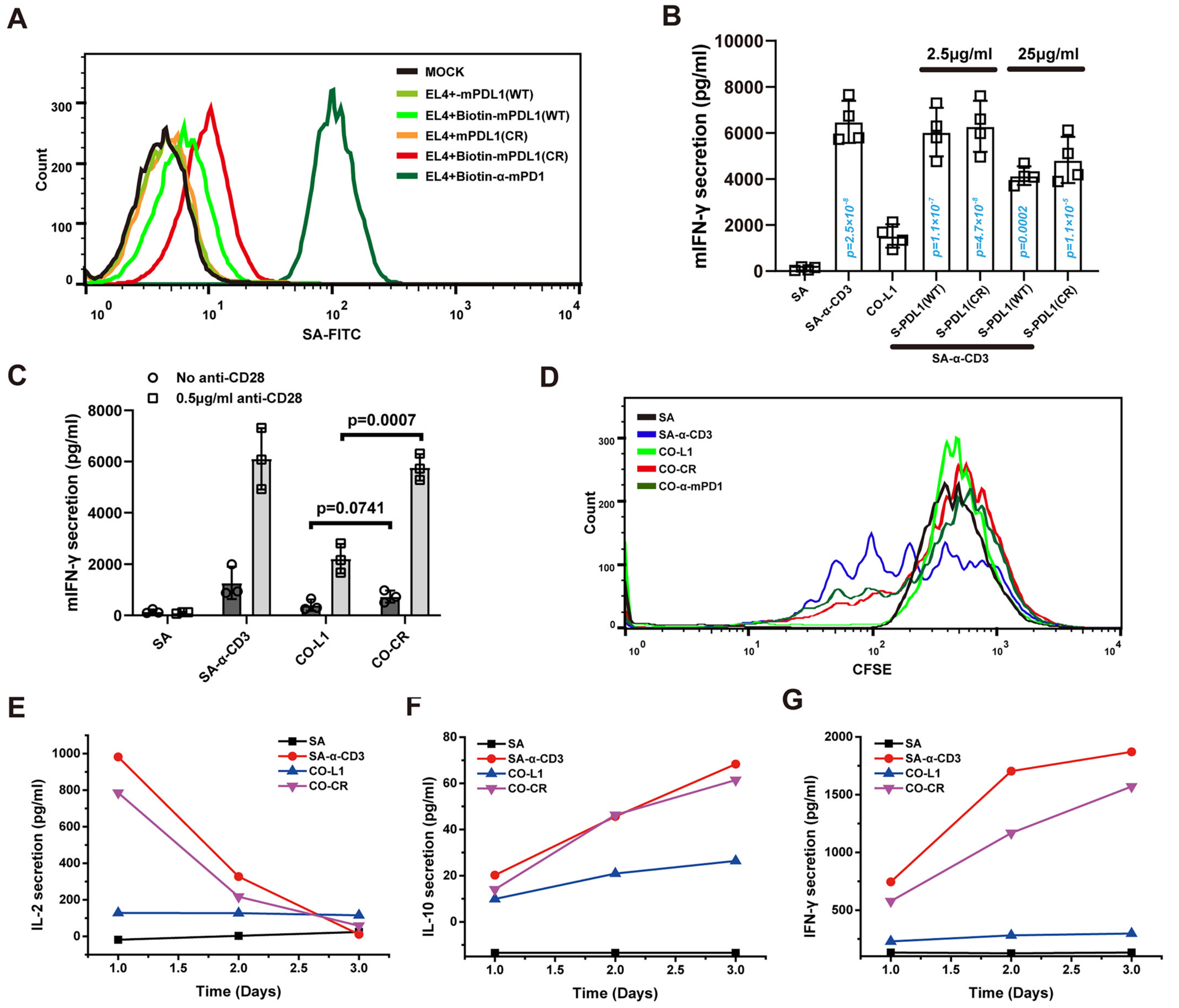

Figure EV6. Soluble mPDL1-CR mutant blocks the inhibitory function of PD-1 in mice.

(**A**) Binding characteristics of wildtype and CR-mutated mouse PD-L1; (**B**) IFN-γ secretion of mouse primary CD8^{+} T cells cocultured with anti-CD3 beads in the presence of mouse S-mPDL1(WT) or mouse S-mPDL1(CR), data were shown as mean ± SD, $n = 4$; (**C**) IFN-γ secretion of mouse primary CD8^{+} T cells stimulated with indicated beads in the presence or absence of mouse CD28 antibody (0.5 μg/ml), data were shown as mean ± SD, $n = 3$; (**D**) Proliferation of primary CD8^{+} T cells stimulated with indicated beads; (**E–G**) Cytokine secretion of mouse CD8^{+} T cells activated with indicated beads. Data information: One-way ANOVA is used to produce *p* value in Fig. EV6B, *t*-test is used to quantify the difference between groups in Fig. EV6C.

 © The Author(s)
